# Enablers of and Barriers to Perinatal Mental Healthcare Access and Healthcare Provision for Refugee and Asylum-Seeking Women in the WHO European Region: A Scoping Review

**DOI:** 10.3390/healthcare12171742

**Published:** 2024-09-01

**Authors:** Kathleen Markey, Mairead Moloney, Catherine A. O’Donnell, Maria Noonan, Claire O’Donnell, Teresa Tuohy, Anne MacFarlane, Susann Huschke, Ahmed Hassan Mohamed, Owen Doody

**Affiliations:** 1 Department of Nursing and Midwifery, Health Research Institute, University of Limerick, V94 T9PX Limerick, Ireland; 2 General Practice and Primary Care, School of Health and Wellbeing, University of Glasgow, 90 Byres Road, Glasgow G12 8TB, UK; 3 School of Medicine, Health Research Institute, University of Limerick, V94 T9PX Limerick, Ireland; 4 Doras, Central Buildings, 51a O’Connell Street, V94 W275 Limerick, Ireland

**Keywords:** perinatal mental health, healthcare access, healthcare provision, refugee women, asylum-seeking women, WHO European Region, scoping review

## Abstract

Perinatal mental health is a growing public health concern. Refugee and asylum-seeking women are particularly susceptible to experiencing perinatal mental illness and may encounter a range of challenges in accessing healthcare. This scoping review sought to identify the enablers of and barriers to healthcare access and healthcare provision for refugee and asylum-seeking women experiencing perinatal mental illness in the WHO European Region. The Preferred Reporting Items for Systematic Reviews and Meta-Analyses extension for Scoping Reviews was applied. Nine databases and six grey literature sources were initially searched in April 2022, and an updated search was completed in July 2023. The search identified 16,130 records, and after the removal of duplicates and the screening process, 18 sources of evidence were included in this review. A data extraction table was used to extract significant information from each individual source of evidence, which was then mapped to the seven dimensions of the candidacy framework. Empirical (*n* = 14; 77.8%) and non-empirical (*n* = 4; 22.2%) sources of evidence were included. The literature originated from seven countries within the WHO European Region, including the United Kingdom (n = 9; 50%), Germany (*n* = 3; 16.7%), Denmark (*n* = 2; 11.2%), Norway (*n* = 1; 5.6%), Greece (*n* = 1; 5.6%), Sweden *(n* = 1; 5.6%), and Switzerland (*n* = 1; 5.6%). The results indicate that, although enablers and barriers were apparent throughout the seven dimensions of candidacy, barriers and impeding factors were more frequently reported. There was also a notable overall lack of reported enablers at the system level. Unaddressed language barriers and lack of attention to the diversity in culturally informed perceptions of perinatal mental illness were the main barriers at the individual level (micro-level) to identifying candidacy, navigating healthcare systems, and asserting the need for care. The lack of culturally appropriate alignment of healthcare services was the key organizational (meso-level) barrier identified. The wider structural and political contexts (macro-level factors), such as lack of funding for consultation time, focus on Western diagnostic and management criteria, and lack of services that adequately respond to the needs of refugee and asylum-seeking women, negatively influenced the operating conditions and wider production of candidacy. It can be concluded that there are multilevel and interconnected complexities influencing access to and provision of perinatal mental healthcare for refugee and asylum-seeking women.

## 1. Introduction

At the end of 2022, there were an estimated 35.3 million refugees around the world, 36% of whom were hosted in Europe [1]. Approximately 630,500 asylum-seeking applications, including 535,000 first-time applications, were recorded in the European Union in 2021, representing an increase of 33% in comparison to 2020 [2]. The United Nations High Commissioner for Refugees defines refugees as ‘persons outside their countries of origin who require international protection because of feared persecution, or a serious threat to their life, physical integrity or freedom in their country of origin as a result of persecution, armed conflict, violence or serious public disorder’. An asylum seeker is defined as ‘any person who is seeking international protection. In some countries, it is used as a legal term referring to a person who has applied for refugee status or a complementary international protection status and has not yet received a final decision on their claim’ [3].

The prevalence of mental illness among refugees and asylum seekers is an area of growing concern. Recent World Health Organization (WHO) estimates put the prevalence of mental illness among conflict-affected populations at 22.1% (which equates to 1 in 5 people), which is much higher than previous estimates [4]. Therefore, providing easily accessible mental healthcare that is responsive to the needs of refugees and asylum seekers is of utmost importance. However, refugees and asylum seekers continue to experience a range of difficulties in navigating general mental healthcare services and receiving the help needed during their resettlement period [5,6,7,8,9].

Approximately 50% of refugees and asylum seekers around the globe are women of childbearing age [10]. Refugee and asylum-seeking women may experience a range of traumatic events before or during their migration journey and resettlement period that increase their susceptibility to developing perinatal mental illness [11,12]. Perinatal mental illness is defined by some scholars as the acute onset, relapse, or recurrence of mental illness during pregnancy or in the year after birth [13]. The World Health Organization expands this definition by distinguishing that women may experience poor mental health for the first time during pregnancy or in the first year following birth, while also identifying that women who are already living with mental illness may experience worsening symptoms [14]. 

Despite the increased policy and practical focus in recent years on improving perinatal mental healthcare, perinatal mental illness among refugee and asylum-seeking women is frequently undiagnosed or untreated [15]. Untreated perinatal mental illness can have devastating effects on the woman, child, and wider family unit [12]. There is a high prevalence of suicidal ideation among refugee women during pregnancy and in the first year after birth [16]. Collectively, this evidence indicates the need for improvements in perinatal mental healthcare access and healthcare provision for refugee and asylum-seeking women.

To date, most systematic literature reviews examining perinatal mental health among refugee and asylum-seeking women report on perinatal mental illness prevalence, particular risk factors, and adverse outcomes for this population [12,14,15,16,17,18,19]. Most notably, an umbrella review of systematic reviews published between 2007 and 2017 reports on the diverse range of difficulties for refugee and asylum-seeking women experiencing perinatal mental health concerns [18]. While these reviews provide an important general overview of perinatal mental health among refugee and asylum-seeking women, they do not examine the specific factors influencing perinatal mental healthcare access and healthcare provision.

Early identification of perinatal mental illness is important in ensuring the prompt implementation of healthcare interventions and treatment. Consequently, the World Health Organization calls for greater integration of perinatal mental healthcare—specifically, screening and treatment services in everyday maternal and child health services [14]. Despite these recommendations, recent reviews report on continued gaps in healthcare delivery for refugee and asylum-seeking women experiencing perinatal mental illness [20,21]. Although these reviews emphasise the need for improvements in perinatal mental healthcare delivery, they do not go far enough in identifying factors that enable and hinder healthcare access across the various stages of the care pathway. Therefore, this scoping review aims to expand on the existing evidence as it specifically examines perinatal mental healthcare access and healthcare provision. It maps the evidence on enablers of and barriers to perinatal mental healthcare across the care pathway, from the perspective of service providers and refugee and asylum-seeking women in the WHO European region, using the candidacy theoretical framework.

The construct of candidacy, as conceptualised by Dixon-Woods and colleagues [22], identifies how eligibility for healthcare is negotiated between individuals and healthcare professionals/healthcare organisations. However, numerous factors influence this dynamic process; therefore, the candidacy journey can be culturally, structurally, professionally, and organisationally constructed and redefined through continuous interactions among individuals, healthcare professionals, and healthcare systems [22]. The candidacy framework suggests that there are seven overlapping stages (identification, navigation, permeability of services, appearances, adjudications, offers, and resistance and operating conditions) in the process of negotiating the candidacy journey [22]. However, suggested refinements to this framework by Mackenzie and colleagues [23] highlight the need for a cyclical as opposed to the staged representation of the candidacy journey, emphasising the complex interplay between micro-level (individual negotiations between service users and healthcare professionals) and wider contextual meso-level (local/organisational) and macro-level (wider societal and political) factors influencing candidacy. This provides a comprehensive view of factors influencing healthcare access, health-seeking behaviour, and acceptance of healthcare interventions, which have been identified as areas that are often overlooked in research about refugee health and broader mental healthcare service provision [6,24]. This framework has successfully been applied to other research examining the candidacy journeys of vulnerable populations such as asylum seekers [25] and those living with complex diseases and illnesses, such as multiple sclerosis [26] and cancer [27]. Therefore, examining the evidence on healthcare access and healthcare provision among refugee and asylum-seeking women living with perinatal mental illness through the lens of candidacy will provide a more comprehensive understanding of the issues.

## 2. Materials and Methods

The primary research question for this review was the following: What are the perspectives of refugee and asylum-seeking women and service providers, with respect to the enablers of and barriers to perinatal mental healthcare access and utilisation within the WHO European Region? A scoping review methodology was selected for this study, as the aim was to identify sources of evidence related to the research question and to map this evidence to the candidacy framework to gain a broad overview of the enablers of and barriers to perinatal mental healthcare. This scoping review was conducted in line with the recommendations by Peters and colleagues [28], which build on previous scoping review guidelines [29,30]. The Preferred Reporting Items for Systematic Reviews and Meta-Analyses extension for Scoping Reviews (PRISMA-ScR) was applied [28], and the PRISMA-ScR checklist [31] is provided in Supplementary File S1. A protocol for this scoping review has been previously published [32].

### 2.1. Eligibility Criteria

The Population, Concept, and Context (PCC) acronym guided the eligibility criteria. Specifically, the population focused on refugee and asylum-seeking women experiencing perinatal mental illness, as well as perinatal mental healthcare service providers. The concept focused on access to and provision of perinatal mental healthcare, and the context focused on perinatal mental healthcare provided within the WHO European Region. Sources of evidence published in English between 1 January 2010 and 7 July 2023, discussing perinatal mental healthcare for refugee and asylum-seeking women within the WHO European Region, from the perspective of either healthcare providers or refugee and asylum-seeking women, were included. In keeping with recommendations by others [33,34] to include grey literature in scoping reviews, all types of literature were included, including non-empirical evidence (e.g., discussion papers, policy papers, editorials, book chapters). Sources of evidence published in languages other than English, before 1 January 2010, referring to perinatal mental healthcare outside the WHO European Region, and which did not focus on perinatal mental health for refugee and asylum-seeking women or did not differentiate their findings between migrant subgroups, even if they included refugees and asylum seekers, were excluded. 

### 2.2. Information Sources and Searches

A search strategy was developed using keywords and synonyms pertaining to refugees and asylum seekers and perinatal mental healthcare. See Supplementary File S2 for an example of a search completed in one database. Nine databases were systematically searched: PsycINFO, Cochrane, Web of Science, MEDLINE, EMBASE, CINAHL Complete, Scopus, Academic Search Complete, and Maternity and Infant Care (OVID). In addition, grey literature searches included Open Grey, Grey Literature Report, Grey Net International, National Institute for Health and Care Excellence, World Health Organization Global Index Medicus, and the TRIP database. All initial searches were performed on 11 April 2022, and an updated search was completed on 7 July 2023.

### 2.3. Selection of Evidence 

Following the removal of duplicates in EndNote 20 (*n* = 2369), the remaining evidence was imported into an online screening software program https://www.rayyan.ai/ accessed on 11 April 2022). The screening was completed independently by five reviewers (K.M., M.N., T.T., C.O.D. and O.D.), firstly by title and abstract, and then by full text. All conflicts were resolved through discussion within the review team. Evidence was included in the review if it met all of the inclusion criteria. Table 1 outlines the definitions for refugees and asylum seekers adopted during screening. The total number of records and reports identified, screened, and included in the review was recorded on a PRISMA flow diagram. 

### 2.4. Data-Charting Process and Data Items

A tabular data-charting Excel document was developed and piloted to determine which variables to extract in ensuring that this review’s aims were achieved. Data on authors, year of publication, title, country of publication, aims, objectives, methodology, data collection and analysis methods (when applicable), population, and population characteristics were included. The seven dimensions of the candidacy framework were also included in the tabular data-charting document. Verbatim data within individual sources of evidence specific to factors influencing access to and provision of perinatal mental healthcare were extracted and mapped to the seven dimensions of the candidacy framework. Others have affirmed the value of examining evidence through the lens of candidacy and recommend using the candidacy framework to map data as a means of explaining the nuances of access to and provision of a range of mental healthcare services [6]. Table 2 outlines the adapted description of the seven overlapping dimensions of candidacy adopted for this review, which guided the data extraction, mapping, and charting. In this way, candidacy was used as a lens to view and extract the data reported in the sources of evidence included in this review (i.e., results and discussion sections of empirical evidence, or the entire piece for non-empirical evidence). 

### 2.5. Synthesis of Results

Descriptive statistics and a narrative summary were used to synthesise the characteristics of the included evidence. Re-occurring patterns of data were collated and summarised using the seven dimensions of the candidacy framework and organised and characterised into enablers of or barriers to receiving and providing perinatal mental healthcare, reflecting an individual’s journey to accessing healthcare, whilst considering the roles that healthcare professionals, health systems, and wider conditions play within this process. A narrative synthesis of each dimension of candidacy is presented in the results, and tables are used to augment the narrative. 

## 3. Results

The search results are presented on the PRISMA flow diagram (Supplementary File S3). Of the 199 full texts screened, 18 sources met the inclusion criteria and were included in this review. The results are organised to present a descriptive overview of the characteristics of the included evidence, and then to describe the enablers of and barriers to perinatal mental healthcare access and provision across the seven dimensions of candidacy. 

### 3.1. Characteristics of Sources of Evidence/Study Characteristics 

Empirical (*n* = 14; 77.8%) and non-empirical (*n* = 4; 22.2%) sources of evidence were included. The literature originated from seven countries within the WHO European Region, including the United Kingdom (*n* = 9; 50%), Germany (*n* = 3; 16.7%), Denmark (*n* = 2; 11.2%), Norway (*n* = 1; 5.6%), Greece (*n* = 1; 5.6%), Sweden (*n* = 1; 5.6%), and Switzerland (*n* = 1; 5.6%) (Table 3).

In the majority of the empirical evidence, the sample involved refugee or asylum-seeking pregnant women or new mothers [36,37,38,39,40,41,42,44,46,47,48]. The five exceptions were studies that included midwives [36], community volunteers [43], health visitors [45], midwives and representatives from key stakeholder groups involved in the perinatal care of refugee women [37], and midwifery students only (no refugee or asylum-seeking women) [42]. Five empirical papers reported the age profile of the women: 18–36 years [40], 22–37 years [41], 19–38 years [39], 18–43 years [47], and 17–32 years [44]. Due to ambiguity in/lack of reporting, it was difficult to ascertain the country of origin of all refugee or asylum-seeking women in the data sources; however, there was representation from Africa, Asia, and Europe (Table 4). This ambiguity was in part due to the undocumented nature of the sample in some studies, and in other cases the women’s country of origin was simply not stated.

### 3.2. Synthesis of Results Using the Candidacy Framework 

In the following sections, findings are mapped under the seven dimensions of the candidacy framework [22], with a focus on identifying the enablers of and barriers to receiving and providing perinatal mental healthcare for women with refugee or asylum-seeking status. There was an overlap of enablers and barriers across the candidacy dimensions, which was expected, as the process of seeking and receiving healthcare is not linear. However, for this review, each dimension of the candidacy framework is presented separately in a descriptive narrative form, with accompanying tabular representation.

#### 3.2.1. Identification of Candidacy: Enablers and Barriers

Identification of candidacy is about recognising health symptoms and knowing when professional attention is needed [22]. Six sources of evidence identified enablers of the identification of eligibility for perinatal mental healthcare for refugee and asylum-seeking women (Table 5). Trusting relationships with friends, peers, or professionals enabled a sense of ease and comfort in openly sharing perinatal mental health experiences and identifying candidacy [43,44,45]. The importance of healthcare professionals and voluntary workers advocating for perinatal mental health needs and supporting refugee and asylum-seeking women in identifying candidacy was also an identified enabler [38,41,47].

Ten sources of evidence identified barriers to the identification of eligibility for perinatal mental healthcare among refugee and asylum-seeking women (Table 5). Barriers that affected refugee and asylum-seeking women in recognising perinatal mental health symptoms and, thus, the need for help, were complex and involved feelings of fear, shame, and stigma [39,40,44,45,48]. These feelings were related to their ‘refugee/asylum seeker’ status, as well as to culturally informed perceptions of mental health and perinatal expectations, with differing perceptions of what was acceptable and what was not; thus, a sense of normalisation of perinatal mental illness symptoms occurred [37,45,48,49]. In some cases, this was further compounded by women’s negative experiences of perinatal healthcare in their countries of origin (e.g., deficient care, dirty hospitals, and corruption), which led to avoidance in recognising the need for help and subsequently seeking help [52]. Language barriers were a challenge when anticipating seeking help, and concerns existed around divulging information using interpreters, regardless of whether the interpreters were friends, family, or professionals [43,46,52].

#### 3.2.2. Navigating Services: Enablers and Barriers

Navigation is about awareness of services and mobilisation of practical resources (e.g., transport, childcare, time off work, financial cost) to facilitate a person in receiving services [22]. Five sources of evidence identified enablers of navigating perinatal mental healthcare services (Table 6). Voluntary workers and people from local communities supported the navigation of perinatal mental healthcare services. Such support was received through one-to-one friendships, local voluntary groups, or church/faith-based community groups [38,41,43]. Signposting to community support services and referrals to healthcare services by healthcare professionals also supported the ease of navigating healthcare services and continuity of follow-up care [47,50].

Six sources of evidence identified barriers to navigating perinatal mental healthcare services (Table 6). Lack of awareness of the healthcare system and the roles of healthcare professionals presented as a barrier that could lead to misconceptions of what to expect [37,50]. Being moved frequently to different geographical locations (e.g., for accommodation reasons) can lead to a loss of trusting relationships, support, and continuity of care with healthcare professionals, resulting in a need to re-navigate new services [38,53]. Language barriers caused difficulties for refugee and asylum-seeking women in navigating services, as awareness and understanding were difficult to achieve with linguistic barriers [37,41,52].

#### 3.2.3. Permeability of Services: Enablers and Barriers

Permeability of services refers to the ‘ease’ with which people can use healthcare services, which takes into consideration the ‘cultural alignment’ between services and the values and needs of service users [22]. Five sources of evidence identified enablers of the permeability of perinatal mental healthcare services (Table 7). Engagement with community voluntary services and the socio-cultural practical support received supports cross-cultural mediation when engaging with healthcare professionals, which nurtures a sense of ease [38,41,43,45,52].

Nine sources of evidence identified barriers to the permeability of perinatal mental healthcare services (Table 7). The lack of cultural alignment of services in meeting the needs of refugee and asylum-seeking women was frequently reported as a barrier to permeable services. Approaches to care delivery that predominately adopted a ‘Western approach’ to perinatal mental healthcare and failed to acknowledge differing cultural norms negatively impacted service permeability—for example, limitations in perinatal mental health screening, over-focus on pharmaceutical interventions, and a lack of approaches to addressing language barriers [36,38,39,40,41,44,46,52]. Healthcare professionals’ lack of competence in optimally responding to the needs of refugee and asylum-seeking women experiencing perinatal mental health concerns also negatively impacts permeability [38,39,42,52].

#### 3.2.4. Appearing at Services and Asserting Candidacy: Enablers and Barriers

Appearance at services is about a person presenting at healthcare services and the actions that they must take to articulate and assert their candidacy and need for care in interactions with healthcare professionals [22]. Three sources of evidence identified enablers of the assertion of candidacy for perinatal mental healthcare services (Table 8). The support provided by community volunteers and the role played by healthcare professionals in advocating for refugee and asylum-seeking women when appearing at services and asserting their candidacy were acknowledged [38,41,43]. 

Four sources of evidence identified barriers to the assertion of candidacy for perinatal mental healthcare services (Table 8). Unaddressed cultural differences and language barriers presented as barriers when appearing at services and asserting candidacy [36,37,39,52]. Gender issues were raised for women whose tradition or religion does not allow interactions with men (i.e., interactions with male healthcare professionals). The busyness of healthcare environments led women to downplay their symptoms, designating themselves as less eligible for care, which meant lost opportunities for discussions with professionals about perinatal mental health and wellbeing [36].

#### 3.2.5. Adjudication by Professionals: Enablers and Barriers 

Adjudication refers to the judgments and decisions made by healthcare professionals that allow or inhibit continued progression through services and further access to care, which may be influenced by operating conditions and resources [22]. Three sources of evidence identified enablers of the judgments and decisions made by healthcare professionals that influenced the progression of perinatal mental healthcare services (Table 9). Witnessing healthcare professionals’ commitment to woman-centred care enabled positive experiences, where women felt seen, heard, and validated [38,40]. Specialist maternity services tailored to refugee and asylum-seeking women were also considered to be supportive [47]. 

Seven sources of evidence identified barriers to the adjudications by healthcare professionals that influenced the progression of perinatal mental healthcare (Table 9). Negative attitudes among healthcare professionals towards refugee and asylum-seeking women were informed by unaddressed prejudices and biases [36,38,39,42]. Fundamentally, the provision of specialist education and training for healthcare professionals in the care of refugee and asylum-seeking women was considered to be a way of negating many negative adjudication experiences [36,37,50,52].

#### 3.2.6. Offers of and Resistance to Services: Enablers and Barriers

Offers and resistance are concerned with the idea that healthcare may be appropriately or inappropriately offered, and that such an offer could be resisted, rejected, or declined by an individual despite their candidacy [22]. Four sources of evidence identified enablers of the offers of and resistance to perinatal mental healthcare services (Table 10). Refugee and asylum-seeking women valued opportunities to engage with healthcare professionals when they felt that they were being understood, which positively informed their decisions about accepting or declining perinatal mental healthcare [40,41,45,50]. Continuity of care helped women to engage with services, as familiarity and trusting relationships could be established [50].

Three sources of evidence identified barriers to the offers of and resistance to perinatal mental healthcare services (Table 10). There was limited evidence in this scoping review of identified candidates resisting, rejecting, or declining care. However, resistance to medication use arose from two data sources, where some women resisted prescribed medication for reasons such as breastfeeding or due to a general lack of understanding about the medication [40,52]. Also of note were midwives’ views in the qualitative study by Firth [36], where they expressed feeling responsible for finding an appropriate service that would accept a referral for a woman with refugee or asylum-seeking status; midwives often ended up using their contacts, networks, and experiences to source appropriate care for women.

#### 3.2.7. Operating Conditions and Local Production of Candidacy: Enablers and Barriers

Operating conditions refer to local specific influences, such as the perceived or actual availability and suitability of resources, and the relationships between service providers and service users, which develop over time through repeated encounters [22]. Two sources of evidence identified enablers of operating conditions and local production of candidacy for perinatal mental healthcare services (Table 11). Having dedicated time during care encounters to engage with women in meaningful and culturally responsive ways is an enabler of the operating conditions of candidacy, as it supports the development of therapeutic relationships [41,45].

Ten sources of evidence identified barriers to operating conditions and local production of candidacy for perinatal mental healthcare services (Table 11). These included an underdeveloped service at the system level, which was not tailored to the needs of women with refugee or asylum-seeker status [36,37,44,46,50]. This was mostly associated with the limitations in resources [37,43,49], wider societal influences [36,39,43], and Western diagnostic and management criteria [36,37,40,52]. A need for more culturally appropriate and sensitive delivery of services that are responsive to the specific needs of refugee and asylum-seeking women was highlighted. Resources and support for healthcare professionals were also highlighted as a necessity for the future development of perinatal mental healthcare services for refugee and asylum-seeking women.

#### 3.2.8. Summary of Findings

The findings of this scoping review were mapped to each stage of the candidacy framework. Table 12 provides a summary of all of the enablers and barriers identified that influence access to and provision of perinatal mental healthcare for refugee and asylum-seeking women in the WHO European Region. Although enablers and barriers were apparent throughout the seven dimensions of candidacy, there was an overall lack of enablers at the system level, which may impact appearance, offers, and local operating conditions.

## 4. Discussion

This review illuminates the interconnected individual-, organisational-, and structural-level factors that influence healthcare access and healthcare provision for refugee and asylum-seeking women experiencing perinatal mental illness in the WHO European Region. Although enablers and barriers were apparent throughout the seven dimensions of candidacy, impeding factors were more frequently reported. Furthermore, there was an overall lack of enablers at the system level, which may then impact the other dimensions of the candidacy journey. At the individual level (micro-level), being understood, developing trusting relationships with healthcare professionals, and the support provided by community volunteers were the main enablers identified. Language barriers and a lack of attention to the diversity in culturally informed perceptions of perinatal mental illness and perinatal mental healthcare were the main barriers to identifying candidacy, navigating healthcare systems, ease of accessing services, and asserting the need for care. The lack of culturally appropriate alignment of perinatal mental healthcare and the limitations of healthcare professionals’ capabilities to respond to the needs of refugee and asylum-seeking women were the key organisational (meso-level) barriers identified. The wider structural and political contextual (macro-level) factors—such as a lack of funding for time in consultations, over-focus on Western diagnostic and management criteria, and a lack of culturally responsive, woman-centred services—negatively influenced the operating conditions and wider production of candidacy. 

The findings from this review echo findings from previous studies that also identified the difficulties experienced by refugee and asylum-seeking women in accessing perinatal mental healthcare [20,21,54,55,56,57]. However, this review adds new insights into the complexities of access to and provision of perinatal mental healthcare for refugee and asylum-seeking women. Incorporating the candidacy framework in the mapping of evidence from the perspectives of service users and healthcare providers helped identify enablers of and barriers to perinatal mental healthcare access and provision that generally receive less focus in the literature or are examined in isolation in individual studies. For example, the individual-level barriers to perinatal mental healthcare access for refugees and asylum-seeking women are acknowledged in the literature (i.e., challenges with accessibility from the perspective of service users) [12,58]. Although this evidence is compelling, it does not sufficiently examine ways of addressing the persistent and systematic barriers that refugee and asylum-seeking women face when engaging with perinatal mental healthcare services. Others have argued for a critical review of services to support refugee and asylum-seeking women experiencing perinatal mental illness [20,21]. This requires a deeper understanding of the complex interactions among refugee and asylum-seeking women, professionals, and systems, as well as how these dynamic relationships are negotiated and navigated across the perinatal mental healthcare journey. Through the lens of candidacy, this study reveals that the challenges in accessing appropriate perinatal mental healthcare services for refugee and asylum-seeking women persist. Moreover, it underscores the broader structural and political factors that have been underexplored in the literature to date. 

Healthcare professionals play a pivotal role in supporting women across the candidacy journey. The World Health Organization [14] calls for the integration of perinatal mental health screening, diagnosis, and management of perinatal mental illness in all healthcare services that provide support to women. However, evidence in the wider literature indicates limitations concerning perinatal mental healthcare capabilities among non-specialist healthcare professionals, i.e., those not specialising in perinatal mental health [59,60]. The findings of this review build on previous work in the field by highlighting how difficulties in accessing perinatal mental healthcare are exacerbated by cultural differences and language barriers, which are often inadequately addressed. Language and culture play a critical role in influencing the candidacy journey for refugee and asylum-seeking women. Although others have highlighted the importance of addressing language barriers in perinatal mental healthcare services [52,61], this review illuminates the poignant impact of systemic unaddressed language barriers. It also highlights how cultural beliefs and values shape culturally informed perceptions of perinatal mental illness and treatments, which contribute significantly to the candidacy journey. While the literature asserts that psychological categories and definitions of perinatal mental health are not universally shared across different populations, including refugee and asylum-seeking women [20,56], these discussions often fail to adequately position this knowledge within the context of perinatal mental healthcare access and provision. The importance of understanding how culture plays a fundamental role in shaping perceptions of pregnancy and perinatal illness, health-seeking behaviour, and expectations of perinatal mental healthcare is acknowledged in the literature [19,20,21,56]. However, the findings of this study indicate that healthcare professionals were not culturally attuned to respond sensitively and appropriately to the perinatal mental healthcare needs of refugee and asylum-seeking women. Although exemplars of good practice were acknowledged, where women felt understood, respected, and adequately supported, most of the evidence reviewed indicated deficiencies in standards of care due to a lack of attention paid to the language barriers and the fact that there are different ways of defining and attending to perinatal mental illness, with the Western psychological approach being only one of them. There is a need to pay greater attention to the diversity of the lived experiences of refugee and asylum-seeking women and the kinds of support and ‘treatment’ that would make sense for them, in addition to traditional psychological/mental health support. Having dedicated time for building trusting relationships during these highly sensitive cross-cultural encounters is paramount whilst negotiating differing culturally informed perceptions and expectations of perinatal mental healthcare. 

Our synthesis indicates a need for better alignment of perinatal mental healthcare services with the lived experiences of refugee and asylum-seeking women who come from diverse cultural and linguistic backgrounds. Other studies have also questioned the cultural appropriateness of perinatal mental healthcare delivery [52,62,63,64,65]. However, the overly medicalised philosophy underpinning perinatal mental healthcare, which prioritises diagnosis and pharmaceutical interventions and assumes that refugee and asylum-seeking women will identify candidacy, is an area that requires further consideration. Similarly, others have identified the need to address the Eurocentric approaches to mental healthcare services more generally [66]. Eurocentrism, in this context, is defined as an approach that ‘emphasises European concerns, culture, and values at the expense of those of other cultures’ [67]. Negotiating treatment plans must take into consideration differing culturally informed conceptualisations of perinatal mental health and associated treatment options, as a means of tackling Eurocentric approaches to perinatal mental healthcare interventions.

This review signals the need for structural changes that embed culturally responsive perinatal mental healthcare in all healthcare policies and practices. Others have asserted the need for focused attention to address the hesitancy in exploring sensitive topics and cultural influences informing perceptions of perinatal mental health [61]. Healthcare systems need a model of perinatal mental healthcare that emphasises inclusivity, the importance of advocacy, and continuity of care and recognises and responds to the lived experiences of refugees and asylum-seeking women. This requires providing readily available resources such as professionally trained interpreters, education and training for healthcare professionals, and targeted support to empower women in recognising perinatal mental healthcare symptoms, seeking help, and addressing some of the barriers to identification of candidacy. The importance of developing refugee- and asylum-seeker-responsive perinatal mental health screening intervention programmes was highlighted, echoing findings from other studies [20,68]. Having input from refugee and asylum-seeking women, as experts with experience in the development of services and authentic engagement with community and voluntary groups, is recommended [69]. This also necessitates a shift from traditional healthcare service delivery mechanisms to more outreach services and social prescribing interventions, facilitated through enhanced collaborations with community and voluntary groups [61]. Social prescribing refers to community provisions that support the health and wellbeing of people living with illness or requiring healthcare—for example, arts-based initiatives or physical group activities [70]. Such social prescribing models have been found to increase self-esteem and empower refugees in seeking healthcare and navigating healthcare systems [71]. 

The lack of empirical evidence exploring access to and provision of perinatal mental healthcare services for refugee and asylum-seeking women in the WHO European Region requires attention. There is a need for further research incorporating implementation science to investigate how culturally responsive perinatal mental healthcare interventions and community-based outreach programs can be developed and implemented across a range of contexts. Incorporating participatory health research to engage refugees and asylum seekers in the research process is important. Others have argued for the need to combine participatory health research and implementation science as a means of accelerating improvements in the cultural responsiveness of healthcare services [69,72]. The lack of empirical evidence about structural influences identified in this review re-affirms the need for further research in this area, as determined by others [20,21]. Some evidence indicates that the length of time spent in the host country is associated with increased levels of health-seeking behaviour, suggesting the need for more longitudinal-type studies [73]. Enhancements in such evidence will inform the development and implementation of necessary perinatal mental health interventions that encourage health-seeking behaviour and are responsive to the perinatal mental health needs of refugee and asylum-seeking women.

### Strengths and Limitations

The use of the updated scoping review method and the incorporation of the candidacy conceptual framework to map the data add some conceptual depth to the analysis of the included sources of evidence, representing key strengths of this review. However, there are also some limitations to be noted. Firstly, only sources of evidence published in English were included, which may have potentially resulted in missing important evidence published in other languages. Secondly, as recommended by the Joanna Briggs Institute and others, critical appraisal and risk-of-bias assessment of evidence was not carried out in this scoping review, as the aim was to map the evidence rather than to provide judgment on its quality [34,74]. Thirdly, as the narrative summary of the results is descriptive, caution is advised when interpreting these results, especially considering the heterogeneity of the included evidence. Finally, data on the country of origin, length of stay in the host country, and residency type were not extracted due to reporting problems in the included papers, and these would be worth close examination in future studies, given that these factors do impact access to and utilisation of healthcare services for refugees and asylum seekers.

## 5. Conclusions

This scoping review illuminates the multilevel and interconnected complexities influencing access to and provision of healthcare services for refugee and asylum-seeking women experiencing perinatal mental illness. At the meso level (organisational level), the configuration of healthcare services and the lack of ‘cultural alignment’ of these services were the key barriers identified. Providing ongoing education and training for healthcare professionals to understand and respect diverse cultural backgrounds, beliefs, and practices related to pregnancy and mental health is a key recommendation. Ensuring that language support services, such as interpreters and translated materials, are readily available to non-native speakers is also a necessity in cultivating a better alignment of perinatal mental healthcare services with the cultural and linguistic needs of women. At the macro level (wider structural and political context), the Eurocentric approaches to service delivery—which predominantly focused on medicalised approaches to treatment, the lack of resources, and refugee- and asylum-seeker-responsive services—negatively influenced the operating conditions and local production of candidacy. Consequently, there is an urgent need to review the perinatal mental healthcare model, which overly focuses on medicalised approaches to treatment. The implementation of policies that promote inclusivity and non-discrimination in healthcare settings is also a key recommendation. Involving refugees and asylum seekers in the development of targeted interventions, aimed at providing refugee- and asylum-seeker-responsive perinatal mental healthcare services that are easily accessible, compassionate, inclusive, respectful, and culturally attuned, is paramount.

## Figures and Tables

**Table 1:** Definitions of refugee and asylum seeker adopted for this review.

Refugee	The United Nations High Commissioner for Refugees defines a refugee as ‘a person who owing to a well-founded fear of being persecuted for reasons of race, religion, nationality, membership of a particular social group, or political opinion, is outside the country of their nationality and is unable to or, owing to such fear, is unwilling to avail himself/herself of the protection of that country’ [35].
Asylum seeker	An asylum seeker is ‘an individual who has applied for asylum on the grounds of persecution in their home country relating to their race, religion, nationality, political belief, or membership of a particular social group. This population remains classified as asylum seeking for as long as the asylum application is pending’ [35].

**Table 2:** Candidacy framework and adapted description of seven dimensions of candidacy.

Dimensions of Candidacy	Adapted Description of Stages
Identification of candidacy	The process by which refugee/asylum-seeking women come to view themselves as suitable candidates for perinatal mental healthcare. The process by which healthcare professionals come to view refugee/asylum-seeking women as suitable candidates for perinatal mental healthcare. 1. How refugee/asylum-seeking women recognise their perinatal mental illness symptoms as needing healthcare services and support. 2. How refugee/asylum-seeking women perceive perinatal mental health and appraise their perinatal mental illness symptoms as requiring help, which legitimises them as candidates for perinatal mental healthcare services. 3. How healthcare professionals identify refugee and asylum-seeking women as candidates for perinatal mental healthcare. 4. How voluntary and charity organisations’ professionals identify refugee and asylum-seeking women as candidates for perinatal mental healthcare.
Navigating services	Knowledge among refugee/asylum-seeking women of perinatal mental healthcare services and how to contact and gain access to these services. 1. The awareness among refugee/asylum-seeking women regarding available support and services for those experiencing perinatal mental health concerns. 2. Knowledge among refugee/asylum-seeking women as to how to contact perinatal mental healthcare services. 3. Appraisal of the practicalities of contacting and accessing perinatal mental healthcare services.
Permeability of services	The ease with which refugee/asylum-seeking women can use perinatal mental healthcare services. 1. The levels of gatekeeping and referral processes/pathways for refugee/asylum-seeking women experiencing perinatal mental concerns. 2. How well configured services are in meeting the specific needs of refugee/asylum-seeking women experiencing perinatal mental health concerns. 3. The degree of ‘cultural alignment’ between refugee/asylum-seeking women and perinatal mental healthcare services (how services align to the cultural/linguistic needs of refugee and refugee/asylum-seeking women).
Appearing at services and asserting candidacy	The actions that refugee/asylum-seeking women must take to assert their candidacy by presenting at services, articulating their issues and their need for care. 1. Factors influencing refugee/asylum-seeking women’s ability to articulate their perinatal mental health concerns and need for care. 2. The work that refugee/asylum-seeking women must do to assert their candidacy and be acknowledged and understood during interactions with healthcare professionals.
Adjudication by professionals	A judgment is made by healthcare professionals that allows or inhibits continued progression of candidacy, and this influences subsequent healthcare access. Processes by which healthcare professionals make decisions that subsequently influence the refugee/asylum-seeking women’s progression through healthcare services and access/eligibility for further perinatal mental healthcare.
Offers of/resistance to services	Offers of perinatal mental healthcare/services are provided by healthcare professionals, which are considered and subsequently accepted or refused by refugee/asylum-seeking women. 1. Factors influencing the type of perinatal healthcare/services offered. 2. The appropriateness and acceptability of offers of perinatal mental healthcare. 3. Factors influencing refugee/asylum-seeking women’s acceptance/rejection of offers for appointments, follow-ups, referral pathways, and perinatal mental health treatment.
Operating conditions and local production of candidacy	Societal and system-level issues that influence perinatal mental healthcare services’ availability and provision for refugee and asylum-seeking women. 1. Factors contributing to the availability and ease of obtaining perinatal mental healthcare services. 2. Factors influencing the availability of resources (i.e., professionally trained interpreters; time) to provide perinatal mental healthcare for refugee and asylum-seeking women. 3. Relational aspects between health professionals and refugee/asylum-seeking women experiencing perinatal mental health concerns (i.e., locally specific influences on interactions during caring encounters).

**Table 3:** Overview description of characteristics of extracted sources.

Variable	No (%)	Author(s) and Year
Data source type	Empirical evidence *n* = 14 (77.8%) Non-empirical evidence *n* = 4 (22.2%)	
Geographical location of data sources	United Kingdom *n* = 9 (50%) Germany *n* = 3 (16.7%) Denmark *n* = 2 (11.1%) Norway *n* = 1 (5.6%) Greece *n* = 1 (5.6%) Sweden *n* = 1 (5.6%) Switzerland *n* = 1 (5.6%)	
Empirical study designs	Qualitative studies *n* = 10 (55.6%)	[36,37,38,39,40,41,42,43,44,45]
	Quantitative studies *n* = 4 (22.2%)	[46,47,48,49]
Non-empirical data sources	Discussion paper *n* = 2 (11.1%)	[50,51]
	Policy paper *n* = 1 (5.6%)	[52]
	Editorial *n* = 1 (5.6%)	[53]

**Table 4:** Countries of origin of refugee or asylum-seeking women.

Africa	Asia	Europe
Ethiopia, Eritrea, Gambia, Ivory Coast, Liberia Sierra Leone, Morocco, Nigeria, Somalia, Somaliland, Sudan, Sierra Leone, Zimbabwe	Afghanistan, Iraq, Palestine, Syria	Albania, Bosnia, Chechnya, Kosovo, Macedonia, Romania, Serbia

**Table 5:** Identification of candidacy: enablers and barriers.

Enablers	Examples from Data Sources
Trusting relationships and advocacy	Feeling like someone cares reduces stress [45]. Support from a befriender/advocate who speaks the same language can help reduce the sense of loneliness, and as a result one may be more likely to reveal difficulties that would otherwise be hidden [43]. A befriender/advocate can listen to experiences and help with signposting appropriate health services [43]. Women valued being a member of a trusted support group [44]. Women shared more about their perinatal experiences in one-to-one settings compared to group settings [44].
Candidacy initiated by others	Professionals picking up on perinatal mental health needs and initiating care [41]. Low-threshold support such as psychosocial walk-in clinics at refugee centres can reduce barriers to perinatal mental health screening and perinatal mental health support [47]. Professional support initiated by social workers, psychologists, and midwives played a significant part in reducing psychological stressors in accessing care [41]. Voluntary workers went out of their way to support women [38].
**Barriers**	**Examples from Data Sources**
Fear and avoidance	Fear of being exposed and vulnerable [40]. Avoiding talking about one’s own circumstances [40]. Sense of loneliness and feeling unable to share one’s plight [40]. Cultural beliefs that talking about oneself may invite the ‘evil eye’ [44]. Unwillingness to attend unfamiliar support groups at unfamiliar premises without a trusted recommendation or companion [44]. Difficulties sharing very personal information in a group whilst claiming asylum [44]. Of the 80 eligible refugee women identified to participate in this study, only 39 (49%) completed the self-reported perinatal mental health questionnaire [48].
Shame and stigma	Loss of independence [40]. Difficulty talking about feelings [40]. Feeling bad when asking for help [45]. The stigma of mental illness in women from diverse cultural backgrounds [39]. Unwillingness to disclose mental health problems in the postpartum period due to stigma of mental illness [39].
Language barriers	Due to linguistic barriers, women may be unwilling or feel unable to seek help [52]. Less likely to disclose information with the use of an interpreter; concerns regarding the lack of confidentiality and lack of code of conduct training for interpreters [43]. Results show that refugee women who resided in Denmark for <5 years faced language barriers, and this may explain why refugee women were less likely to engage in perinatal mental health screening [46].
Perceptions of perinatal mental health	Self-rated perceptions of health are varied [37]. Different perspectives and expectations on how postpartum health and wellbeing should be experienced [37]. Lack of understanding and awareness of postpartum depression [49]. Instead of identifying as ‘sick’, women would express a need for practical help and support instead [49]. A significant association was seen between migration status and lack of perinatal mental health screening. There are more barriers to perinatal mental health screening for refugee women compared to Danish-born women [46]. Poor experiences in their country of origin led to avoiding maternity care as a refugee or asylum seeker in a new country, e.g., deficient care, dirty hospitals, and corruption where women only receive care if money is offered to staff [52]. Of the 39 respondents, only 3 (7.7%) were assessed as having a possible perinatal mental illness. This unexpectedly low result might reflect a difference in expectations among the refugee women of how the postpartum period should be experienced [48].

**Table 6:** Navigating services: enablers and barriers.

Enablers	Examples from Data Sources
Community support	A specialist antenatal support group run by the voluntary sector provided the opportunity to gain support from peers [38]. A local church was a place of support and an opportunity to meet people from the same background/country [38]. Women appreciated when people from the local community reached out with support [41]. Volunteers helped refugee and asylum-seeking women to access health services, including maternity care and mental health service support [43].
Referrals by healthcare professionals	Clarifying healthcare professionals’ roles and responsibilities, clarifying their influence on immigration proceedings, and clarifying their role in helping access to free care can increase a woman’s engagement with services [50]. Healthcare professionals referred women to specialist services; 74% of the refugee women were advised to undergo outpatient psychotherapy after transferring to municipal accommodation because of their severe mental health problems [47]. Professionals providing psychosocial walk-in clinics for pregnant refugees and new mothers referred patients to other care providers, including specialists, midwives, mother–child facilities, support offers provided by churches, and counselling and outreach centres, as well as charitable organisations such as the German Caritas Association, when necessary [47].
**Barriers**	**Examples from Data Sources**
Lack of awareness of the healthcare system	Lack of knowledge of the role of healthcare professionals (e.g., midwives) and lack of knowledge of the healthcare system [50]. Unrealistic expectations of the health system due to misinformation or excessive optimism, resulting in disappointment [37]. Lack of understanding of the policies and capacity of the country of destination [37].
Moving locations	Moving location (e.g., due to accommodation issues) meant losing social support and established relationships with healthcare workers such as midwives, GPs, or doulas [38]. Negative impact of moving during pregnancy, leading to loss of social support and continuity of care with healthcare professionals [53]. Women must relearn how to navigate health services in the new location [53].
Language barriers	It is very difficult to navigate healthcare services when you do not speak the language [41]. Due to linguistic barriers, women are often not aware of the services available to them [53]. Language as a barrier to perinatal mental healthcare [37].

**Table 7:** Permeability of services: enablers and barriers.

Enablers	Examples from Data Sources
Cultural mediation support	Engagement with other services helps with understanding [45]. Engagement with community support and voluntary groups supports cultural mediation [38,41]. Practical and social support is particularly beneficial [52]. Support from community volunteers is valued [43]. Engagement initiated by healthcare professional [41].
**Barriers**	**Examples from Data Sources**
Lack of cultural alignment of services	Limitations to approaches to perinatal mental health screening [46,52]. Lack of meaningful engagement with healthcare professionals [36]. Perceptions that women do not meet Western diagnostic criteria [36]. Services lack empathy and compassion for the lived experience of refugee and asylum seekers [36]. Discriminatory attitudes of staff [36]. Over-focus on pharmaceutical interventions [52]. Experiencing neglectful care encounters [36,40]. Prioritising physical care at the detriment of emotional care [36]. Not being understood [38,40]. Unaddressed language barriers [36,39,41]. Women raised issues of safety and a lack of trust in healthcare professionals [44].
Capabilities of healthcare professionals	Feelings of intimidation during care encounters [38]. Healthcare professionals’ prejudices [42]. Healthcare professionals’ over-focus on cultural differences [42]. Ill-prepared healthcare professionals [52]. Lack of awareness among HCPs of cultural and religious factors that influence healthcare expectations [39].

**Table 8:** Appearing at services and asserting candidacy: enablers and barriers.

Enablers	Examples from Data Sources
Advocacy support	Healthcare professionals initiating professional support and perinatal mental healthcare [41].
	Supported by community volunteers [38,43].
	Engagement with community support and voluntary groups supports ease in appearing at services [38,41].
**Barriers**	**Examples from Data Sources**
Lack of attention to cultural differences	Cultural or religious notions may lead to perceiving healthcare as unacceptable when not given by a same-sex care provider [39].
	Gender was a barrier for women whose tradition or religion does not allow interactions with men [37].
	There were cultural barriers in the relationships between women and healthcare professionals, with a lack of understanding of culture and traditions related to pregnancy and birth [37].
Language barriers	Language barriers with women compromised their ability to articulate their needs [36].
	The language barrier posed a particular problem [39].
	Difficulty in articulating needs [52].
Busyness of services	The busyness of services and healthcare professionals meant a lack of time spent with women, so less opportunity to share their mental health worries [36].
	Woman did not want to bother midwives with their problems, as they appeared so busy [36].
	Woman downplayed their symptoms, so as not to bother the busy healthcare professionals, with a sense of being less eligible for care [36].

**Table 9:** Adjudication by professionals: enablers and barriers.

Enabler	Examples from Data Sources
Commitment to woman-centred care	Women felt respected, acknowledged, ‘seen’, and ‘heard’ by the midwife [40]. Midwives come to your rescue and care for women [38].
Specialist services for refugee and asylum-seeking women	Specialist services at detention centres (in collaboration with maternity care services) support women with perinatal mental health concerns [47].
**Barriers**	**Examples from Data Sources**
Negative attitudes towards refugees and asylum seekers	Intimidation by social workers implying that they would take children from women [38]. Intimidation implying that women were not entitled to public funds [38]. Healthcare professionals lack cultural awareness of cultural factors that influence perinatal mental healthcare expectations [39]. Healthcare professionals’ prejudices and biases [36,42].
Ill-prepared healthcare professionals	Complex factors (e.g., the reason for migration, social situations, and cultural constructions of mental health) can present challenges for midwives who care for vulnerable migrant women [50]. Lack of preparation among healthcare professionals, with training, preparation, and education inadequate for supporting pregnant migrant and refugee women [52]. Minimal investment in the interdisciplinary and interprofessional training of the perinatal mental healthcare team [37]. Midwives reported that women are seen as one-dimensional by other professionals, with migrant status superseding mental health needs [36].

**Table 10:** Offers of and resistance to services: enablers and barriers.

Enablers	Examples from Data Sources
Being understood	Being understood by the healthcare professional [40]. Engaging in meaningful discussions about perinatal mental health [50]. Culturally competent healthcare professionals [41,45]. Continuity of care is more likely to lead to a trusting relationship and the ability to have sensitive discussions [50].
**Barriers**	**Examples from Data Sources**
Over-focus on pharmacological interventions	Prescribed medicines that cannot be taken due to breastfeeding [40]. Women present with somatic symptoms and tend to prefer practical help instead of pharmacological interventions [52].
Difficulty finding appropriate services for referral	Midwives expressed feeling responsible for finding a service that would accept a referral for a woman with refugee or asylum-seeking status; midwives often ended up using their personal contacts, networks, and experiences to source appropriate care offers for women [36].

**Table 11:** Operating conditions and local production of candidacy: enablers and barriers.

Enablers	Examples from Data Sources
Dedicating time	Structure, extra time, and training in cultural competence helped in understanding and building trust with refugee and asylum-seeking women [45]. Dedicated time during care encounters [45].
**Barriers**	**Examples from Data Sources**
Lack of appropriate services	Services not suitable for refugee and asylum-seeking women, who would therefore be less likely to discuss their mood or disclose information [36]. Lack of screening for refugee women compared to women living in their country of origin [46]. Culturally appropriate services, with high cultural understanding and informed consent practices, are needed [37]. Referral numbers for asylum-seeking mothers into perinatal mental health services are low. Sensitive service models and referral mechanisms responsive to the specific needs of vulnerable people are needed [44]. Lack of service integration and continuity of care [37]. If healthcare professionals lack cultural awareness and knowledge, then cultural misunderstandings and conflicts occur [49].
Lack of funding for time in consultations	There is evidence of the need to develop relationship-centred interventions with refugee and asylum-seeking mothers and their infants, and this requires resources in terms of time [44]. Pregnant asylum-seeking and refugee women often have complex health and social care needs that midwives may have difficulty in meeting due to the resources needed in terms of managing workloads and limitations of time [43]. Low engagement during crisis—healthcare professionals’ burnout due to workload [37]. Limitations of allocated resources result in a lack of time to meet the healthcare needs of women in a culturally appropriate manner [37].
Western diagnostic and management criteria	Western diagnostic criteria used for acceptance into general perinatal mental health services may not be suitable for refugee or asylum-seeking women [36]. Refugee women are less likely to engage in perinatal mental health screening [46]. Doctor-centred and patriarchal systems of care [37]. Focus on pharmaceutical interventions [40,52].
Wider societal influences	Midwives were cautious about who they sought support or guidance from, due to the discriminatory attitudes of other staff members [36]. The asylum seeker is seen as different, and the difference is often expressed negatively. A general prejudice against asylum seekers [39]. Poor attitudes and a lack of understanding of their needs [43]. Fear of financial charges for healthcare [50]. Migration status supersedes perinatal mental health needs [36].

**Table 12:** Summary of enablers and barriers in the candidacy, stages 1–7.

	Identification	Navigation	Permeability	Appearances at Health Services	Adjudication	Offers and Resistance	Operating Conditions and the Local Production of Candidacy
Enablers	Trusting relationships and advocacy Candidacy initiated by others	Community support Referrals by healthcare professionals	Cultural mediation supports	Advocacy and support	Commitment to women-centred care Specialist services for refugee and asylum-seeking women	Being understood	Dedicating time
Barriers	Fear and avoidance Shame and stigma Language barriers Perceptions of perinatal mental health	Lack of awareness of the healthcare system Moving locations Language barriers	Lack of cultural alignment of services Capabilities of healthcare professionals	Lack of attention to cultural differences Language barriers Busyness of services	Negative attitudes towards refugees and asylum seekers Ill-prepared healthcare professionals	Over-focus on pharmacological interventions Difficulty providing appropriate services for referral	Lack of appropriate services Lack of funding for time in consultations Western diagnostic and management criteria Wider societal influences

## Data Availability

The original contributions presented in this study are included in the article/Supplementary Materials; further inquiries can be directed to the corresponding author.

## Supplementary Materials

The following supporting information can be downloaded at https://www.mdpi.com/article/10.3390/healthcare12171742/s1: Supplementary File S1: Preferred Reporting Items for Systematic Reviews and Meta-Analyses extension for Scoping Reviews (PRISMA-ScR) checklist; Supplementary File S2: Sample of one database search; Supplementary File S3: PRISMA flow diagram.

## References

[1] United Nations High Commissioner for Refugees (UNHCR) Global Trends Report 2022. https://www.unhcr.org/ie/media/global-trends-report-2022#:~:text=At%20the%20end%20of%202022,events%20seriously%20disturbing%20public%20order.

[2] (2022). Eurostat. Asylum Statistics. Statistics Explained. https://ec.europa.eu/eurostat/statistics-explained/index.php/Asylumstatistics.

[3] United Nations High Commissioner for Refugees (2020). Asylum-Seekers. https://www.unhcr.org/asylum-seekers.html.

[4] Charlson F., van Ommeren M., Flaxman A., Cornett J., Whiteford H., Saxena S. (2019). New WHO prevalence estimates of mental disorders in conflict settings: A systematic review and meta-analysis. Lancet.

[5] Satinsky E., Fuhr D.C., Woodward A., Sondorp E., Roberts B. (2019). Mental health care utilisation and access among refugees and asylum seekers in Europe: A systematic review. Health Policy.

[6] van der Boor C.F., White R. (2020). Barriers to Accessing and Negotiating Mental Health Services in Asylum Seeking and Refugee Populations: The Application of the Candidacy Framework. J. Immigr. Minor. Health.

[7] Blackmore R., Boyle J.A., Fazel M., Ranasinha S., Gray K.M., Fitzgerald G., Misso M., Gibson-Helm M. (2020). The prevalence of mental illness in refugees and asylum seekers: A systematic review and meta-analysis. PLoS Med..

[8] Gewirtz A.H., Muldrew L., Sigmarsdóttir M. (2022). Mental health, risk and resilience among refugee families in Europe. Curr. Opin. Psychol..

[9] Nowak A.C., Namer Y., Hornberg C. (2022). Health Care for Refugees in Europe: A Scoping Review. Int. J. Environ. Res. Public Health.

[10] United Nations High Commissioner for Refugees (2019). What is a Refugee?. https://www.unhcr.org/what-is-a-refugee.html.

[11] Stevenson K., Fellmeth G., Edwards S., Calvert C., Bennett P., Campbell O.M.R., Fuhr D.C. (2023). The global burden of perinatal common mental health disorders and substance use among migrant women: A systematic review and meta-analysis. Lancet Public Health.

[12] Almeida L.M., Moutinho A.R., Siciliano F., Leite J., Caldas J.P. (2024). Maternal Mental Health in Refugees and Migrants: A Comprehensive Systematic Review. J. Int. Migr. Integr..

[13] O’Hara M.W., Wisener K.L. (2014). Perinatal mental illness: Definition, description and aetiology. Best Pract. Res. Clin. Obstet. Gynaecol..

[14] World Health Organization (2022). Guide for Integration of Perinatal Mental Health in Maternal and Child Health Services.

[15] Ganann R., Sword W., Newbold K.B., Thabane L., Armour L., Kint B. (2020). Influences on mental health and health services accessibility in immigrant women with post-partum depression: An interpretive descriptive study. J. Psychiatr. Ment. Health Nurs..

[16] Fellmeth G., Nosten S., Khirikoekkong N., Oo M.M., Gilder M.E., Plugge E., Fazel M., Fitzpatrick R., McGready R. (2021). Suicidal ideation in the perinatal period: Findings from the Thailand-Myanmar border. J. Public Health.

[17] Bradby H., Humphris R., Newall D., Phillimore J. (2015). Public Health Aspects of Migrant Health: A Review of the Evidence on Health Status for Refugees and Asylum Seekers in the European Region.

[18] Heslehurst N., Brown H., Pemu A., Coleman H., Rankin J. (2018). Perinatal health outcomes and care among asylum seekers and refugees: A systematic review of systematic reviews. BMC Med..

[19] Sharma E., Howard N., Duclos D. (2020). Navigating new lives: A scoping review and thematic synthesis of forced migrant women’s perinatal experiences. J. Migr. Health.

[20] Firth A., Haith-Cooper M., Dickerson J., Hart A. (2022). Perinatal depression: Factors affecting help-seeking behaviours in asylum seeking and refugee women. A systematic review. J. Migr. Health.

[21] Ramadan M., Rukh-E-Qamar H., Yang S., Vang Z.M. (2023). Fifty years of evidence on perinatal experience among refugee and asylum- seeking women in Organization for Economic Co-operation and Development (OECD) countries: A scoping review. PLoS ONE.

[22] Dixon-Woods M., Cavers D., Agarwal S., Annandale E., Arthur A., Harvey J., Hsu R., Katbamna S., Olsen R., Smith L. (2006). Conducting a critical interpretive synthesis of the literature on access to healthcare by vulnerable groups. BMC Med Res. Methodol..

[23] Mackenzie M., Conway E., Hastings A., Munro M., O’Donnell C. (2013). Is ‘Candidacy’ a useful concept for under-standing journeys through public services? A critical interpretive literature synthesis. Soc. Policy Adm..

[24] Liberati E., Richards N., Parker J., Willars J., Scott D., Boydell N., Pinfold V., Martin G., Jones P.B., Dixon-Woods M. (2022). Qualitative study of candidacy and access to secondary mental health services during the COVID-19 pandemic. Soc. Sci. Med..

[25] Chase L.E., Cleveland J., Beatson J., Rousseau C. (2017). The gap between entitlement and access to healthcare: An analysis of “candidacy” in the help-seeking trajectories of asylum seekers in Montreal. Soc. Sci. Med..

[26] Pétrin J., Finlayson M., Donnelly C., McColl M.A. (2021). Healthcare access experiences of persons with MS explored through the Candidacy Framework. Health Soc. Care Community.

[27] Tookey S., Renzi C., Waller J., von Wagner C., Whitaker K.L. (2018). Using the candidacy framework to understand how doctor-patient interactions influence perceived eligibility to seek help for cancer alarm symptoms: A qualitative interview study. BMC Health Serv. Res..

[28] Peters M.D.J., Marnie C., Tricco A.C., Pollock D., Munn Z., Alexander L., McInerney P., Godfrey C.M., Khalil H. (2020). Updated methodological guidance for the conduct of scoping reviews. JBI Évid. Synth..

[29] Arksey H., O’Malley L. (2005). Scoping studies: Towards a methodological framework. Int. J. Soc. Res. Methodol..

[30] Levac D., Colquhoun H., O’Brien K.K. (2010). Scoping studies: Advancing the methodology. Implement. Sci..

[31] Tricco A.C., Lillie E., Zarin W., O’Brien K.K., Colquhoun H., Levac D., Moher D., Peters M.D.J., Horsley T., Weeks L. (2018). PRISMA Extension for Scoping Reviews (PRISMA-ScR): Checklist and Explanation. Ann. Intern. Med..

[32] Markey K., MacFarlane A., Noonan M., Moloney M., Huschke S., O’donnell K., O’Donnell C., Tuohy T., Mohamed A.H., Doody O. (2022). Service user and service provider perceptions of enablers and barriers for refugee and asylum-seeking women accessing and engaging with perinatal mental health care services in the WHO European region: A scoping review protocol. Int. J. Environ. Res. Public Health.

[33] Godin K., Stapleton J., Kirkpatrick S.I., Hanning R.M., Leatherdale S.T. (2015). Applying systematic review search methods to the grey literature: A case study examining guidelines for school-based breakfast programs in Canada. Syst. Rev..

[34] Pollock D., Davies E.L., Peters M.D.J., Tricco A.C., Alexander L., McInerney P., Godfrey C.M., Khalil H., Munn Z. (2021). Undertaking a scoping review: A practical guide for nursing and midwifery students, clinicians, researchers, and academics. J. Adv. Nurs..

[35] United Nations High Commissioner for Refugees (UNHCR) (2017). Master Glossary of Terms. https://www.unhcr.org/glossary/#refugee/#asylum-seeker.

[36] Firth A. (2021). Developing Effective Maternity Services for Refugee and Asylum Seeking Women with Symptoms of Perinatal Depression: Increasing the Visibility of an Invisible Population. https://pure.hud.ac.uk/en/publications/developing-effective-maternity-services-for-refugee-and-asylum-se.

[37] Papadakaki M., Iliadou M., Sioti E., Petelos E., Vivilaki V. (2021). The Perinatal Journey of a Refugee Woman in Greece: A Qualitative Study in the Context of the ORAMMA Project to Elucidate Current Challenges and Future Perspectives. Sexes.

[38] Ellul R., McCarthy R., Haith-Cooper M. (2020). Destitution in pregnancy: Forced migrant women’s lived experience. Br. J. Midwifery.

[39] Henry J., Beruf C., Fischer T. (2020). Access to Health Care for Pregnant Arabic-Speaking Refugee Women and Mothers in Germany. Qual. Health Res..

[40] Barkensjö M., Greenbrook J.T.V., Rosenlundh J., Ascher H., Elden H. (2018). The need for trust and safety inducing encounters: A qualitative exploration of women’s experiences of seeking perinatal care when living as undocumented migrants in Sweden. BMC Pregnancy Childbirth.

[41] Gewalt S.C., Berger S., Ziegler S., Szecsenyi J., Bozorgmehr K. (2018). Psychosocial health of asylum seeking women living in state-provided accommodation in Germany during pregnancy and early motherhood: A case study exploring the role of social determinants of health. PLoS ONE.

[42] Haith-Cooper M., Bradshaw G. (2013). Meeting the health and social needs of pregnant asylum seekers, midwifery students’ perspectives: Part 1; dominant discourses and midwifery students. Nurse Educ. Today.

[43] Mccarthy R., Haith-Cooper M. (2013). Evaluating the impact of befriending for pregnant asylum seeking and refugee women. Br. J. Midwifery.

[44] O’Shaughnessy R., Nelki J., Chiumento A., Hassan A., Rahman A. (2012). Sweet Mother: Evaluation of a pilot mental health service for asylum-seeking mothers and babies. J. Public Ment. Health.

[45] Castaner M.M., Villadsen S., Poulsen V., Nørredam M. (2021). How to promote maternal mental wellbeing in refugee mothers through home visiting: The Danish experience. Eur. J. Public Health.

[46] Castaner M.M., Hvidtfeldt C., Villadsen S.F., Laursen B., Pedersen T.P., Norredam M. (2022). Disparities in postpartum depression screening participation between immigrant and Danish-born women. Eur. J. Public Health.

[47] Kaufmann C., Zehetmair C., Jahn R., Marungu R., Cranz A., Kindermann D., Friederich H., Bozorgmehr K., Nikendei C. (2022). Maternal mental healthcare needs of refugee women in a State Registration and Reception Centre in Germany: A descriptive study. Health Soc. Care Community.

[48] Løvlie A.L., Madar A.A. (2017). Postpartum Depression Among Somali Women in Norway. J. Immigr. Minor. Health.

[49] Ratcliff B.G., Sharapova A., Suardi F., Borel F. (2015). Factors associated with antenatal depression and obstetric complications in immigrant women in Geneva. Midwifery.

[50] Firth A.D., Haith-Cooper M. (2018). Vulnerable migrant women and postnatal depression: A case of invisibility in maternity services. Br. J. Midwifery.

[51] Haith-Cooper M., McCarthy R. (2015). Striving for excellence in maternity care: The Maternity Stream of the City of Sanctuary. Br. J. Midwifery.

[52] Iliadou M., Papadakaki M., Sioti E., Giaxi P., Leontitsi E., Petelos E., Muijsenbergh M.V.D., Tziaferi S., Mastroyiannakis A., Vivilaki V.G. (2019). Addressing mental health issues among migrant and refugee pregnant women: A call for action. Eur. J. Midwifery.

[53] Feldman R., Musgrave A. (2015). “Dispersal” of asylum seeking women in UK during pregnancy. BMJ.

[54] Tobin C.L., Di Napoli P., Beck C.T. (2018). Refugee and Immigrant Women’s Experience of Postpartum Depression: A Meta-Synthesis. J. Transcult. Nurs..

[55] Watson H., Harrop D., Walton E., Young A., Soltani H. (2019). A systematic review of ethnic minority women’s experiences of perinatal mental health conditions and services in Europe. PLoS ONE.

[56] Haque S., Malebranche M. (2020). Impact of culture on refugee women’s conceptualization and experience of postpartum depression in high income countries of resettlement: A scoping review. PLoS ONE.

[57] Griswold K.S., Vest B.M., Lynch-Jiles A., Sawch D., Kolesnikova K., Byimana L., Kefi P. (2021). “I just need to be with my family”: Resettlement experiences of asylum seeker and refugee survivors of torture. Glob. Health.

[58] Verschuuren A., Tankink J., Franx A., van der Lans P., Erwich J., Jong E.F., de Graaf J. (2023). Community midwives’ perspectives on perinatal care for asylum seekers and refugees in the Netherlands: A survey study. Birth.

[59] Noonan M., Galvin R., Jomeen J., Doody O. (2019). Public health nurses’ perinatal mental health training needs: A cross sectional survey. J. Adv. Nurs..

[60] Webb R., Uddin N., Ford E., Easter A., Shakespeare J., Roberts N., Alderdice F., Coates R., Hogg S., Cheyne H. (2021). Barriers and facilitators to implementing perinatal mental health care in health and social care settings: A systematic review. Lancet Psychiatry.

[61] Das R., Beszlag D. (2021). Paternal Experiences of Perinatal Loss: An Evidence Review Commissioned by the National Childbirth Trust.

[62] Fair F., Raben L., Watson H., Vivilaki V., van den Muijsenbergh M., Soltani H. (2020). Migrant women’s experiences of pregnancy, childbirth, and maternity care in European countries: A systematic review. PLoS ONE.

[63] Ikhilor P.O., Hasenberg G., Kurth E., Asefaw F., Pehlke-Milde J., Cignacco E. (2019). Communication barriers in maternity care of allophone migrants: Experiences of women, healthcare professionals, and intercultural interpreters. J. Adv. Nurs..

[64] Fair F., Soltani H., Raben L., van Streun Y., Sioti E., Papadakaki M., Burke C., Watson H., Jokinen M., Shaw E. (2021). Midwives’ experiences of cultural competency training and providing perinatal care for migrant women a mixed methods study: Operational Refugee and Migrant Maternal Approach (ORAMMA) project. BMC Pregnancy Childbirth.

[65] Markey K., Noonan M., Doody O., Tuohy T., Daly T., Regan C., O’donnell C. (2022). Fostering collective approaches in supporting perinatal mental healthcare access for migrant women: A participatory health research study. Int. J. Environ. Res. Public Health.

[66] Bansal N., Karlsen S., Sashidharan S.P., Cohen R., Chew-Graham C.A., Malpass A. (2022). Understanding ethnic inequalities in mental healthcare in the UK: A meta-ethnography. PLoS Med..

[67] Ilia X., Sangeeta R., Schwarz H., Berlanga J.L.V., Moreiras A., Shema A.K. (2016). Eurocentrism and Orientalism. The Encyclopedia of Postcolonial Studies.

[68] Willey S.M., Gibson M.E., Blackmore R., Goonetilleke L., McBride J., Highet N., Ball N., Gray K.M., Melvin G., Boyd L.M. (2024). Perinatal mental health screening for women of refugee background: Addressing a major gap in pregnancy care. Birth.

[69] MacFarlane A., Huschke S., Marques M.J., Gama A., Kinaan W., Hassan A., Papyan A., Phelan H., Severoni S., Kumar B. (2024). Normalising participatory health research approaches in the WHO European region for refugee and migrant health: A paradigm shift. Lancet Reg. Health Eur..

[70] Cooper M., Avery L., Scott J., Ashley K., Jordan C., Errington L., Flynn D. (2022). Effectiveness and active ingredients of social prescribing interventions targeting mental health: A systematic review. BMJ Open.

[71] Zhang C.X., Wurie F., Browne A., Haworth S., Burns R., Aldridge R., Zenner D., Tran A., Campos-Matos I. (2021). Social prescribing for migrants in the United Kingdom: A systematic review and call for evidence. J. Migr. Health.

[72] Markey K., Macfarlane A., Manning M. (2023). Time to re-envisage culturally responsive care: Intersection of participatory health research and implementation science. J. Adv. Nurs..

[73] Markova V., Sandal G.M., Pallesen S. (2020). Immigration, acculturation, and preferred help-seeking sources for depression: Comparison of five ethnic groups. BMC Health Serv. Res..

[74] Aromataris E., Lockwood C., Porritt K., Pilla B., Jordan Z. (2020). JBI Manual for Evidence Synthesis.

