# Supplementary figures and images for: Enablers of and Barriers to Perinatal Mental Healthcare Access and Healthcare Provision for Refugee and Asylum-Seeking Women in the WHO European Region: A Scoping Review
Source: Healthcare (Basel). 2024 Sep 1;12(17):1742. doi: 10.3390/healthcare12171742 (PMC11395031; doi:10.3390/healthcare12171742)

### Supplementary File 3: PRISMA flow Chart

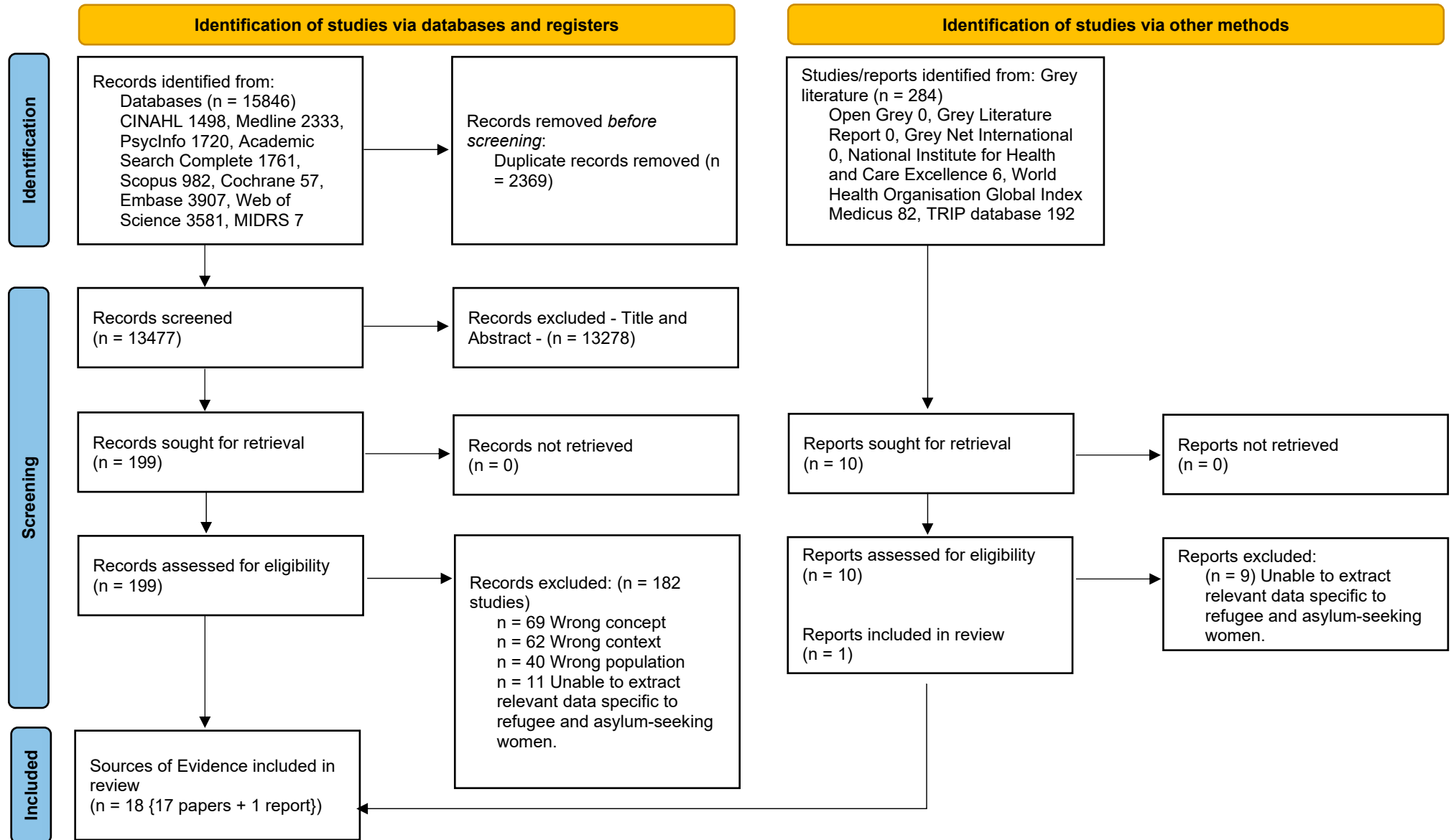

Supplement: Supplementary file 1 [file healthcare-12-01742-s001.zip › Supplementary file 3 PRISMA PMH.pdf]
